# Ferret: a sentence-based literature scanning system

**DOI:** 10.1186/s12859-015-0630-0

**Published:** 2015-06-20

**Authors:** Padmini Srinivasan, Xiao-Ning Zhang, Roxane Bouten, Caren Chang

**Affiliations:** Computer Science, University of Iowa, Iowa City, IA USA; Department of Biology, St Bonaventure University, St Bonaventure, NY USA; Department of Cell Biology and Molecular Genetics, University of Maryland, College Park, MD USA

**Keywords:** Gene-centric relationships, Sentence retrieval, Text retrieval, Sentence ranking, Scientific workflow

## Abstract

**Background:**

The rapid pace of bioscience research makes it very challenging to track relevant articles in one’s area of interest. MEDLINE, a primary source for biomedical literature, offers access to more than 20 million citations with three-quarters of a million new ones added each year. Thus it is not surprising to see active research in building new document retrieval and sentence retrieval systems. We present Ferret, a prototype retrieval system, designed to retrieve and rank sentences (and their documents) conveying gene-centric relationships of interest to a scientist. The prototype has several features. For example, it is designed to handle gene name ambiguity and perform query expansion. Inputs can be a list of genes with an optional list of keywords. Sentences are retrieved across species but the species discussed in the records are identified. Results are presented in the form of a heat map and sentences corresponding to specific cells of the heat map may be selected for display. Ferret is designed to assist bio scientists at different stages of research from early idea exploration to advanced analysis of results from bench experiments.

**Results:**

Three live case studies in the field of plant biology are presented related to *Arabidopsis thaliana*. The first is to discover genes that may relate to the phenotype of open immature flower in Arabidopsis. The second case is about finding associations reported between ethylene signaling and a set of 300+ Arabidopsis genes. The third case is on searching for potential gene targets of an Arabidopsis transcription factor hypothesized to be involved in plant stress responses. Ferret was successful in finding valuable information in all three cases. In the first case the bZIP family of genes was identified. In the second case sentences indicating relevant associations were found in other species such as potato and jasmine. In the third sentences led to new research questions about the plant hormone salicylic acid.

**Conclusions:**

Ferret successfully retrieved relevant gene-centric sentences from PubMed records. The three case studies demonstrate end user satisfaction with the system.

## Background

As of 2013 there were more than 20 million citations indexed in MEDLINE^1^ compiled from about 5600 journals. Along with PubMed its public interface, MEDLINE supported close to 2.5 billion searches in 2013 alone. Since 2010 the annual increase in number of searches has been from 14 % to 23 %. The problem is that the size and growth rate of publications (about 750,000 citations added/year)^2^ make it difficult for researchers to track relevant literature even in their own specialties let alone in related disciplines. Knowledge isolation in the biosciences can be risky especially as research is increasingly interdisciplinary. As a solution to this problem a variety of document ranking and retrieval systems have been developed [1–3] to augment NLM’s PubMed system. A second type of solution to counter the literature tracking challenge is through sentence retrieval [4–6]. Because sentences are at a finer grain they impose a lower overhead in terms of reading; yet they can still lead the scientist to relevant documents. Our goal is to present Ferret a system for retrieving sentences indicating gene-centric relationships. Ferret is specialty independent as long as genes/proteins are the focus. Besides being gene-centric Ferret supports multi-species searches, is transparent, and maintains a memory of user jobs. It employs document filters and gene ambiguity detection and resolution strategies, and measures of sentence interestingness.

In developing Ferret our goal is to support bio scientists with varying literature-tracking goals. A scientist may want to find out all that is known about two interacting genes, or to explore possible functions and phenotypes of a newly encountered gene, or find explanations for observed empirical results, or find elaborations on observations extracted from public databases such as OMIM [7]. Ferret emphasizes flexibility in supporting these and other information needs. The only expectation is that the user is interested in exploring a gene/protein-centered relationship. The system is not confined to specific types of relationships, as is generally the case with information extraction systems that preprocess MEDLINE data. Ferret is a retrieval system to support literature scanning where the user may be interested in any number of genes for any types of relationships. We also assume that while the user may be investigating a specific species, information published in the context of other species can be valuable. This has implications in how we disambiguate between different text appearances of a string representing the gene of interest.

Our goal is also to have Ferret support scientists at various stages of research. For example a user may have a hunch about a gene function. If this is early stage research the user may only be willing to invest a small, limited amount of time to finding and reading relevant literature. Displaying sentences about gene function independent of species seems a reasonable strategy. Similarly while scanning a technical paper a user might become intrigued by one or more mentioned genes in the context of a phenotype. Instead of shelving this curiosity the scientist may peruse a sentence-level summary of connections produced by Ferret. In expression studies, a scientist may have identified several hundred to thousands of genes with interesting expression patterns associated with a specific treatment condition. Through a single job Ferret may be used to find relevant sentences that indicate connections between the genes and the treatment. And Ferret retains memory of jobs so that retrieved sentences (and linked documents) may be reviewed and/or refined later. In essence, Ferret supports efficient scanning of large swaths of literatures to follow hunches, find confirmatory evidence, build plausible stories explaining relationships observed, build/assess hypotheses etc. The importance of literature based evidence for several stages of research is indicated by others such as [8].

In summary, we present Ferret, our sentence-based retrieval system designed for bioscience researchers interested in scanning the literature to explore gene-centric relationships. We present Ferret’s design and three live case studies demonstrating its use.

### Related work

The thriving subfield of bioscience information extraction relates to our work. The general goal is to extract and optionally normalize bioscience entities and their relationships [9–11] from MEDLINE records and from full-texts. Recent reviews provide excellent summaries and pointers to the literature [12, 13]. Extraction is done to serve a variety of higher-level goals such as gene annotation [14] to find protein-protein interactions [10], answer questions automatically [15], event extraction [16] and augment public datasets such as MINT [17] and UniProtKB/Swiss-Prot. Special research initiatives such as BioCreAtiVe, TREC and CLEF have contributed significantly to research on information extraction methods exploring natural language processing algorithms and machine learning methods [10, 18–20]. While Ferret is a retrieval system and not an information extraction system, it faces similar challenges in gene recognition and ambiguity resolution. In order to stay flexible and as current as PubMed data, Ferret retrieves records and processes them at run time. In comparison information extraction systems usually focus on specific entities and relationships of interest and pre-process the documents to extract them. Ferret is also designed to work directly with end users involving them (optionally) in steps such as query expansion and disambiguation.

A variety of document retrieval systems offer access to MEDLINE and allied databases. Research initiatives such as Biocreative [14], TREC genomics [21] and BioASQ [22] have helped shape core technologies for document retrieval in the biomedical domain. This review highlights the variety of approaches implemented. GoPubMed [1] interfaces to MEDLINE through the Gene Ontology. Retrieved documents are organized using the GO terms identified in them. Key to the approach is the term extraction algorithm for matching text phrases and the structurally constrained GO terms. RefMed [2] exploits user relevance feedback to refine ranking of the retrieved documents. It uses RankSVM for supervised ranking. When the retrieved set is large then RefMed [2] uses sampling strategies to learn the ranking function from feedback. MEDLINERanker [3] is also a ‘supervised’ system as the user provides a training set of relevant documents as input. Noun usage in this set is compared with usage in a background dataset. Discriminative words are identified and then used to rank the retrieved documents. A system close to ours in spirit is Quertle [6] offering semantic searching of relationships. By pre-processing the text collection they have extracted about 300 million relationships, based on sentence diagrams of subject-verb-object relations. A query composed of one or more concepts is searched in this relationship collection producing ‘focused’ results. The query is also searched directly as keywords and these documents retrieved are shown under ‘broader’ results. It is in this aspect that Quertle and Ferret are similar. Both the focused and broader results are displayed under separate tabs. Additionally Power Terms™ may be used in the query. These are general classes of search terms, e.g., diseases, enzymes and proteins. Quertle appears well suited to search for a pair of concepts or concept classes at a time.

A ranking of retrieved documents can still be hard to peruse efficiently. Sentence retrieval is an option worth exploring to make this more efficient. iHOP pre-computes a navigable network of gene/protein sentences from MEDLINE that is updated daily. When a search is conducted for a gene of interest iHOP displays the sentences in which it appears. Mentions of other genes in these sentences may then be clicked to view their sentence lists. Sentences are ranked, for example those with experimental evidence are ranked higher. Verbs indicating associations also influence sentence ranking [4]. The focus appears to be on a single or a pair of genes at a time rather than a list of genes. Textpresso offers full-text, sentence retrieval from the literature of model organisms. Searches are sophisticated and may involve categories such as genes of a specific species and Gene Ontology classes [5]. (This facility appears similar to the power terms of Quertle). While several species-specific search systems are offered Textpresso does not support searches across species.^3^ CoPUB [23] is designed to help in the analysis of a single gene, pairs of genes or a set of genes. For the given input it identifies co-occurring concepts in the literature classified into categories such as disease, drug, GO term, pathway, tissue and metabolite. When given a set of genes it calculates literature-based keyword enrichment. Enrichment is calculated using the Fisher exact test, in which the extent of co-publications of a given keyword with a set of regulated genes (in MEDLINE) is statistically tested against a background set. The system is limited to human, mouse and rat genes. Chilibot [24] is another search engine that looks for sentences about relationships between terms on a list and between members of two lists of terms. In this aspect it is similar to Ferret. It returns results as a graph. MedEvi is another system to show the relationships between two concepts [25]. Its key characteristic is that it uses the KWIC concordance, a lexicographic tool, as a display device. It also supports a user adjustable distance threshold between the concepts based on the reasonable assumption of proximity being a marker of semantic relatedness. MEDIE [26] also emphasizes concept relations. It uses sentence parsing to extract relations from MEDLINE in the form of predicate argument structures involving genes, proteins and diseases with id based links to relevant ontologies. The user’s query is converted into a region algebra expression that can be used to search for predicates satisfying user criteria. MEDIE may be used to answer structured queries such as ‘What activates p53?’. Both MedEvi and MEDIE limit searches to one relationship at a time, though MedEvi seems agnostic to relationship type.

There is also a class of sentence-based systems directly targeting hypothesis discovery that stems from pioneering literature-based discovery work by Swanson [27]. FACTA+ and EVEX database are two examples. With FACTA+ a user may explore direct and indirect associations between concepts such as genes and diseases with special emphasis on biomolecular events such as gene activation [28]. The EVEX database is another example of a rich sentence based resource for biomolecular events to study associations between genes and proteins [29]. Given an input gene, the system will retrieve sentences indicating events as well as a set of generally relevant sentences. By browsing the retrieved lists one can also look for interactions such as between gene pairs. Both rely on prior information extraction to identify entities and events. Neither supports inputs in the form of two lists of concepts of unlimited size.

Hypothesis discovery systems directly target the generation of new ideas. Ferret’s goal is in essence literature scanning, a user-driven-process in which hypothesis discovery is only one possible outcome (clarification and elaboration of existing ideas, finding confirmatory evidence are other possible outcomes). Ferret is designed to handle two lists of unrestricted size as input, a list of genes and a list of keywords. Keywords could be GO terms, treatments, chemicals, diseases or any other concept of interest. It then retrieves sentences pertaining to all *pairs* of genes and to gene-keyword *pairs*. Although CoPUB can handle many genes at a time, it is to explore keyword overexpression for the group as a whole while we focus on pairwise relationships. Chilibot looks similar to Ferret in that it takes two lists of terms and looks for sentence relationships between members of one list and between pairs from different lists. Ferret does not extract semantic categories of information (enzymes, cells, function etc.) or relationships as in information extraction systems. It stays keyword-based but is augmented by synonym expansion, gene ambiguity resolution, species identification and sentence ranking. This is intentional so as to keep the system lightweight, as current as PubMed, and relevant to gene-based information needs in general. Staying as general as ‘keywords’ in the second list allows maximum flexibility in satisfying user information needs. Ferret also allows cross species searches, and maintains the notion of a job with memory.

## Methods

### System design

Ferret is a prototype gene-centric sentence retrieval system designed for scientists to quickly and efficiently scan large swaths of the bioscientific literature. The system takes as input a list of one or more genes of interest (GoI) and an optional list of keywords. There is in theory no limit to the size of the lists. The job is accepted and completed offline. The user receives intimation when the job has been completed. Keywords may be a combination of phenotypes, treatments, drugs, function verbs or any other category of information. Ferret outputs a set of ranked sentences with links to the associated documents. Sentences are selected if they are likely to show a relationship between two genes or between a gene and a keyword. As mentioned earlier the system is designed to be useful at different stages of the research process. If the research is at an initial stage then the GoIs and keywords may be identified from some prior work. GoIs can also be identified at more advanced research stage as for example from the outcomes of gene expression experiments to find explanations for observed expression patterns. Ferret has been designed to satisfy the following requirements.

1. Emphasize Relationships: The retrieval system should support the exploration of relationships. These could be between pairs of genes or between genes and keywords. ‘Keywords’ may be used to specify molecular and biological functions, phenotypes, treatments, diseases, drugs, and any other phrase of interest.
2. Jobs: The system should be able to process and retain memory of user jobs (defined by an input set of genes and keywords of interest). Users should be able to revisit and refine their jobs at later points in time.
3. Multi Species Perspective: While the user may focus on a particular species, the system should allow for exploration of related information in other species.
4. Transparency: The system should readily show why a text chunk is retrieved.
5. Rating: The system should allow the user to rate sentences for relevance and use this for evaluation.
6. Intuitive Display of Results: Given that a job may involve thousands of genes and many keywords, the system should display results in a manner that minimizes cognitive load.
7. Broad Applicability: The system should assist scientists with different interests (e.g., a scientist studying genes related to human obesity or a scientist studying genes related to agriculturally important plant traits. The common denominator is the study of relationships involving genes/proteins.
8. The system should be as current as MEDLINE in the underlying data.
9. The system should give users control of their jobs. Specifically, it should be easy to modify input job details (e.g., add or delete genes, their synonyms or homologs, keywords and keyword synonyms).

### System schema & description

**Fig. 1:**
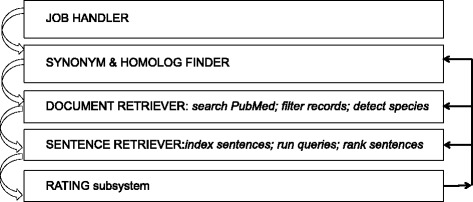
System components. The figure shows processing sequence with the arrows on the left side. The user may modify aspects of the job (homologs, synonyms etc.) at several points as shown by arrows on the right

Ferret has five components as shown in Fig. 1. The user provides a list of genes and a second list of keywords as input. The components shown process this input in sequence.

**Fig. 2:**
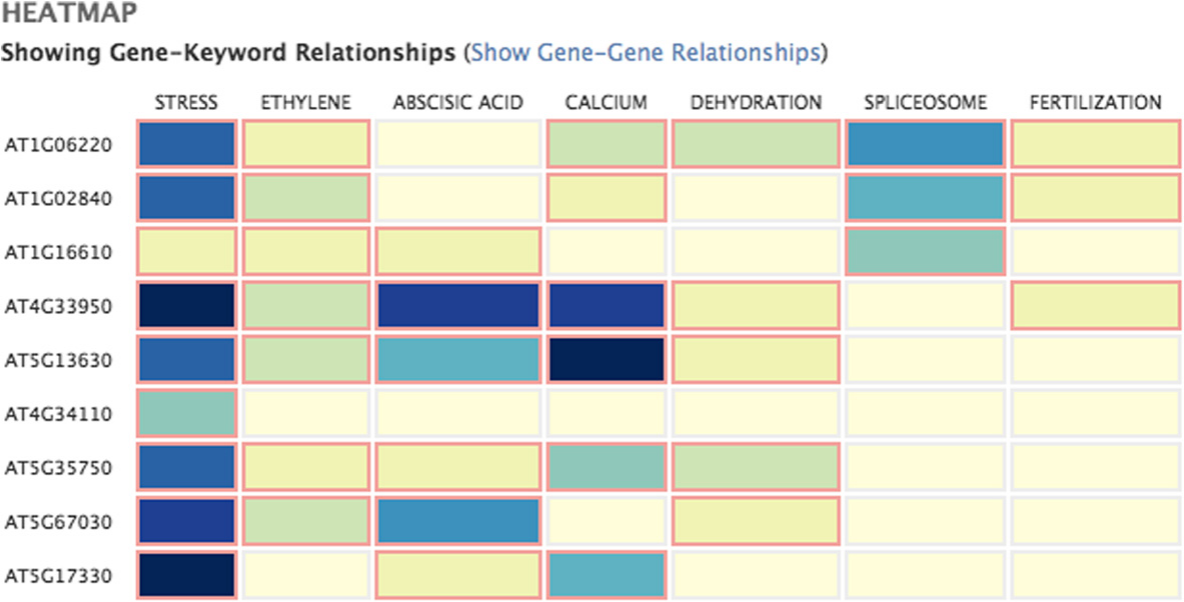
Sample heat map output given nine genes and seven keywords

**Fig. 3:**
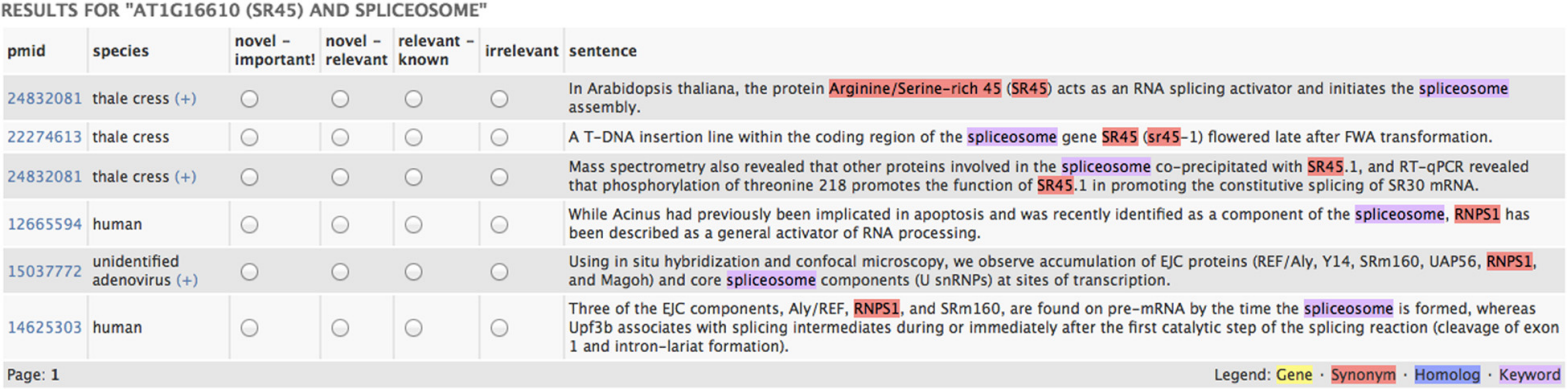
Sample sentence output for a heat map cell. The display also shows PubMed Identifiers (pmid), species identified in the records and relevance rating options

1. Job Handler: This administrative component creates and tracks new jobs. Jobs are tied to specific users and may be revisited. A user may access an administration front end to examine gene synonyms, homologs, retrieved documents and reject any of them. More genes and keywords may be added as well. The user may also opt out of using homologs.
2. Synonym and Homolog Finder. Gene names, synonyms and symbols are harvested through public resources such as Entrez Gene. Sometimes MEDLINE offers little published information on a gene therefore Ferret offers the option to expand the search using its homologs as determined from NCBI’s HomoloGene.^4^
3. Document Retriever. Documents are retrieved using PubMed APIs. This is done separately for each gene in the input set. Because of ambiguities in gene names and symbols we filter the retrieved documents. Most of the logic for gene name disambiguation is as used in our earlier research [30]. Our goal is to make sure that the retrieved record is about a gene. We apply an additional document filter using the Genia Tagger.^5^ Specifically we remove documents in which fewer than seven instances of tagged entities (proteins, DNA, RNA, cell line, cell type) are found. This threshold was determined from a 5-fold cross-validation experiment (with F-score of 0.70) using a collection of approximately 6400 documents that had about 2000 relevant items about some gene and the rest were not relevant. This is a general filtering strategy intended to retain documents that are about genes. The strategy may be augmented by using other tagging services such as BANNER.^6^ Genia Tagger was chosen because of its performance and its ease of use. We also detect the species discussed in each document. This is done using Linnaeus [31] that has been used successfully by others and in our own research [32].
4. Sentence Retriever. Retrieved documents are split into sentences using the Genia Sentence Splitter [33] and indexed using Indri [34]. A separate sentence index is created for each gene. We use each index to retrieve sentences showing connections with the other genes and keywords of the input set. This allows us to explore sentences for all gene-gene and gene-keyword pairs. Indri ranks sentences by retrieval score. We filter out sentences that are too small to be meaningful (less than 5 words) and re-arrange the remaining list so that sentences from the same document are blocked together (and positioned at their best rank).
5. Rating subsystem: The user may provide a relevance rating for each sentence. Ratings are recorded in a backend database and used for system evaluation. Essentially a user may select one of the following ratings for a sentence: (a) relevant (b) maybe relevant and (c) not relevant.^7^
6. Result display: A job may involve thousands of genes and many keywords. Since we retrieve results for every gene – keyword and gene – gene pair this can yield a large set of sentences. Therefore looking at a pairwise, linear listing of results can be inefficient. A heat map is an intuitive device to present such results and one that is already familiar to bio scientists. A heat map both partitions the result space usefully and provides a good visual overview. In Ferret darker cell colours indicate greater prevalence of retrieved sentences that might indicate a relationship between the row and column objects. There are two heat maps, one for gene – keyword results and the other for gene – gene results. The heat map display allows the user to distinguish between object pairs in prevalence of retrieved sentences. For example, a gene that has interacting sentences with many of the given keywords can easily be spotted. Similarly a keyword that has interacting sentences with many of the input genes can readily be identified. Figure 2 shows a sample heat map for a set of nine genes and seven keywords. The sentences shown below the grid are the top ranking ones for the cell selected by the user. Figure 3 shows the sentences retrieved for the pairing of gene SR45 and keyword spliceosome. Genes, synonyms, homologs and keywords in a sentence are colour coded. At this point, if for example a synonym or a homolog is incorrect, these may be deleted through the administration front end to the system. This same front end may also be used to modify the input set of genes and keywords.

## Results and discussion

### Case studies

We demonstrate the use of Ferret with three case studies involving different research goals.

### Case 1. Discoveries related to open immature flower phenotype

Professor Zhang’s group is interested in understanding the phenotype of Open Immature Flower (OIF) in an *sr45-1* enhancer mutant of *Arabidopsis thaliana*. This research may be viewed as early stage research on understanding OIF. SR45 is a RNA binding protein and an activator of pre-mRNA splicing. In a genetic screen for factors contributing to the fertility of the *sr45-1* mutant, the group identified *kin11* mutation as a genetic enhancer of the *sr45-1* mutation. All biological tests confirmed that *kin11* is a true genetic enhancer of *sr45-1*. However, there was no obvious connection between SR45 and KIN11. Zhang was unfamiliar with the KIN11 gene. This case presents the process of discovering more about the pathway relating AtKIN11 and SR45 with assistance from Ferret.

The group’s first goal was to look for established working models on SR45, KIN11 and their potential interactions, in different organisms (plants, animals and fungi). The aim was to quickly find a small group of papers to read. This is where Ferret had a key role. Ferret was used to run a PubMed query using all synonyms and other commonly used names of KIN11. Ferret retrieved 27 PubMed documents. Since this was a small number of documents their full texts were manually collected (mostly from PubMed Central) and these were processed for relevant sentences using Ferret. Keywords specified were activation, binding, signaling, interaction, expression, metabolism, response, phosphorylation, transcription and localization. Sentences containing the gene and one or more keywords were retrieved. A quick scan through the sentence set alerted Zhang that the bZIP family of genes (and KIN10, an Arabidopsis homolog of KIN11) occurred quite frequently. For example there were 12 sentences that had both bZIP and KIN10 with the keyword transcription. Ferret was used to retrieve the 38 sentences having both bZIP and KIN10 from the 27 documents and these gave further clues indicating the importance of the bZIP family. The following is an example with bZIP and the keyword ‘metabolism’ (PMID 19716759):

*“The****bZIP****transcription factors activate the transcription of G-box containing target genes involved in amino acid****metabolism****, energy consumption, and gluconeogenesis* [24, 27] *to reprogram metabolism, here exemplified by the ProDH2 and ASN1 genes shown to be direct tar …”.*

This example and similar sentences drew Zhang’s attention to two other targets: *ProDH2* and *ASN1*. It was known that when two classes of bZIPs interact with each other, it leads to transcription of *ProDH2* and *ASN1*. It was also known that SR45 affects the expression of *ASN1* under glucose treatment [35]. After testing the expression of *ASN1* in the *sr45-1* enhancer mutant, Zhang’s group found that SR45 and AtKIN11 have a synergistic effect on the RNA level of *ASN1.*

Zhang’s group decided to pursue bZIPs further. Although their experiments did not show a change in the RNA level of bZIPs or KIN10/KIN11, they hypothesized that these genes are more likely affected at their protein level because KIN10 and KIN11 are kinases that are activated by phosphorylation, bZIPs are phosphorylation targets of activated KIN10/KIN11 and *ASN1* is a direct target of bZIPs. And so their research continues.

The sentences displayed by Ferret quickly brought the focus of Zhang’s research to bZIP and its target, ASN1. Formal rating for each sentence was not provided. Instead Zhang gave overall impressions indicating benefits such as time saved for literature collection and information sorting. Essentially it shortened the time required for Zhang’s group to further their understanding of the relationship between KIN11 and ASN1. Ferret is now being used to explore other possible connections between KIN11 and SR45 on a literature-based exploration. The research completed has led to a poster presentation at Rustbelt RNA meeting in October, 2013 [36] and a paper has been published [37] with attribution to our system.

### Case 2. Discoveries related to ethylene hormone signaling

Our second case is from a collaboration with Professor Chang on a set of 304 proteins of interest. This case was conducted more formally (compared with case 1) with attention paid to user ratings, number of retrieved sentences etc. Prof. Chang studies ethylene hormone signaling in the model plant species *Arabidopsis thaliana*. Chang carried out a proteomic study in Arabidopsis to identify proteins that change in abundance after 3 h of treatment with the hormone ethylene. From this study, she identified 304 proteins that either accumulated or decreased significantly after ethylene treatment. A 2011 paper reports her findings [38] and her research with this data continues. While the dataset presented a useful resource for understanding ethylene signaling, the project presented a common challenge in making use of a large data set in the –omics era. The challenge was to determine which of the identified proteins (and/or their associated genes) were already known to be associated with ethylene hormone responses and which had not been previously linked with ethylene or with any previously identified proteins in ethylene hormone signaling. Chang and her colleagues had carried out this task through literature searches “by hand”, but they believed that many potential connections were likely missed, since this was an impossible task to carry out consistently and in a reasonable amount of time. Moreover, they believed there could be hidden or non-obvious connections between protein subsets, and that these protein subsets were probably not previously linked with ethylene. Having such insight would greatly enhance the value of the dataset, both advancing their knowledge of ethylene hormone signaling and providing new avenues for further investigations. With several of their objectives in mind we used Ferret to see if we could find sentences presenting relationships between any of these proteins and ethylene phenotypes (e.g., proliferation of root hairs and induction of fruit ripening). Input to this job consisted of the 304 proteins and ethylene as a keyword.

Ferret retrieved 389 sentences from 264 unique PubMed documents. Since this was a large set of documents, we limited processing to the MEDLINE records obtained from PubMed. Chang classified 38 (10 %) sentences as relevant, 28 (7 %) as promising but difficult to judge with confidence and the remaining 323 (83 %) were considered non relevant. 29 of the 38 relevant sentences were further declared to provide *excellent confirmation of, support for and extension of their mass spectrometry results* reported in [38] and the remaining 9 relevant sentences *exhibit clear solid connections with ethylene*. The following sentences are examples for the first category of relevant sentences.

Example 1) PMID: 16662588 (1982 publication), Species: Potato (*Solanum tuberosum*). *“The amount of lipoxygenase associated with mitochondria increased when tubers were treated with ethylene.”*

Example 2) PMID: 19334761 (2009 publication), Species: Kiwifruit (*Actinidia deliciosa). “Lipoxygenase gene expression in ripening kiwifruit in relation to ethylene and aroma production.”*

Example 3) PMID: 22002747 (2011 publication); Species: Norway spruce (*Picea abies*) *“Jasmonate and ethylene are likely to both be involved in formation of traumatic resin ducts based on elevated transcripts of genes encoding lipoxygenase and 1-aminocyclopropane-1-carboxylic acid oxidase associated with resin duct formation.”*

Chang expressed great interest in examples 1 through 3 because they showed previously published correlation between ethylene treatment and an increase in lipoxygenase in 3 other species. These supported and strengthened the findings in their mass spectrometry experiment in Arabidopsis. They had not mentioned the potato, kiwifruit and Norway spruce findings in their 2011 paper, because they were not aware of these results; also the annotation for the lipoxygenase gene, AT1G55020, did not say anything about ethylene. Examples 4 and 5 fall into the second category of sentences that help to confirm and strengthen the ethylene connections in Chang’s mass spectrometry results. Most of the examples were from different stages or other parts of the plant, and some were from other species, which helped again to extend their mass spectrometry results. These results would have been included if their paper had been written now. Example 4 is for gene PhyA (AT1G09570) (several such sentences from different documents were retrieved for this gene) while example 5 is for gene RHD1 (AT1G64440).

Example 4) PMID: 16805726 (2006 publication) Species: Pea (*Pisum sativum*). *“Mutant phyA phyB plants produce significantly more ethylene than WT plants, and application of an ethylene biosynthesis inhibitor rescued many aspects of the phyA phyB mutant phenotype.”*

Example 5) PMID: 14973760 (2004 publication) Species: Arabidopsis (*Arabidopsis thaliana*). *“We find that ethylene specifically suppresses all visible aspects of the rhd1 phenotype.”*

Chang also identified a third category of 28 sentences that looked promising, however further information was needed to make a firm decision.

In summary, Ferret helped this Arabidopsis research group do a broad scan of the literature related to 304 proteins by retrieving 389 sentences (from 264 documents) each linking at least 1 protein to ethylene. There were 38 excellent sentences and 28 promising ones in this set. These were easy to identify in the set particularly when compared to the traditional approach of manually finding such information from PubMed. A majority of the non-relevant sentences (280/323) were retrieved because one of the 304 proteins, the ethylene biosynthesis gene AtACO2, is always associated with the word “ethylene” in virtually every publication. These sentences were easy to eliminate as non-relevant. Some non-relevant sentences were retrieved due to the occurrence of the chemical ethylene glycol, used in non-plant biology related papers. The 38 relevant sentences were from 25 documents and these referenced 26 species totally. Ten of the 25 documents referred to species other than Arabidopsis. These provided 14 of the 38 relevant sentences. Overall, the relevant sentences (and their documents) provided Chang’s group with insight into the underlying molecular mechanisms of ethylene responses and build plausible stories to explain their observations. These also opened new avenues for wet bench studies in Chang’s laboratory to further investigate the connections that the study has revealed. Besides contributing to Chang’s study of genes related with ethylene phenotypes, this case provided key observations for Ferret. Specifically, confirmatory evidence obtained from literature on other species can be valuable. At least some of this evidence could have been useful at the time of writing their research paper. This strengthens our intuition that Ferret may also be useful at more advanced stages of research.

### Comparison with other systems with case 2

We use case 2 to compare Ferret with other systems. The systems we selected are Quertle, Chilibot, Textpresso and MEDIE. We selected the first three, as they are similar in spirit to Ferret. While MEDIE is an example of a current system it does not allow inputs in the form of lists. Our goal was to see how one might use these systems to process the dataset of 304 proteins of case 2 and to what degree one might be successful at finding the relevant sentences.

As said earlier Quertle is the closest in spirit to Ferret in that it allows searching of concept pairs and the system allows one input to be a class of objects via their notion of Power Terms™. Most related to case 2 is their $Proteins Power Term (general class of proteins) and the three specific Power Terms™: $ProteinKinases or $ProteinPhosphatases or $ProteinReceptors. Unfortunately these groups are far larger than the set of 304 proteins of interest in our case and to the best of our knowledge Quertle does not allow restricting the Power Terms™ to a particular subset. Searching on $Proteins AND ethylene retrieves 297 sentences under focused results and 6742 under broader results. Focused results are obtained from a pool of sentences identified as containing proteins through a pre-processing information extraction step. Inferring from the highlights in the display for broader results, these are documents where the keywords: protein or proteins and ethylene co-occur. Examining the 297 focused results we note that Quertle finds only 1 of the 25 relevant PubMed documents that Ferret found. It is possible that the remaining 24 documents are in the set of 6742 broader results, but this set is clearly too large for an end user scientist to peruse effectively. Moreover, the system does not allow us to check this as it limits downloads to the first 1000 results.

Chilibot offers the facility to search for links between two lists of terms. Similar to Ferret, terms in list 1 would be pairwise searched and also searched pairwise with list 2 entries. List 2 entries would only be searched paired with list 1 entries. This would have suited us ideally in that we could regard the set of 304 proteins as list 1 and place the single keyword of interest (ethylene) in list 2. Unfortunately Chilibot is limited to 50 terms in list 1 which reduces the scope of the case study considerably. Furthermore despite several tries we did not receive any output even when we reduced list 1 to 49 terms. Chilibot successfully provided output when the input was limited to just 2 to 3 proteins at a time.

The third system compared against is Textpresso, which offers searchable indices for twenty-four different literatures. Some of these literatures are species specific while others are for example disease centric (HIV, cancer). Ferret retrieved 38 relevant sentences from 25 documents referencing 26 species. The only species from this set indexed in Textpresso is Arabidopsis and we found 15 of the relevant documents. Examples of other species referenced by our relevant sentences are Norway spruce, kiwi fruit, sugar beet and mango. Ten documents in the 25 (40 %) did not reference Arabidopsis. These provided 14 of the 38 (37 %) relevant sentences. It would not be possible to find these relevant sentences via Textpresso. A key additional point is that we had to execute the searches manually for each gene – keyword pair. This is because Textpresso does not allow a custom list of genes as input in combination with a keyword.

MEDIE was the fourth system tried. The gene was entered as one concept and ethylene was entered as the second concept. The relation was left unspecified as for the other systems. Despite trying each synonym for the gene we were only able to retrieve 4 of the 25 relevant documents.

Table 1 summarizes the comparison of the three systems that returned results with Ferret. A limitation in this comparison is that for practical reasons we have not explored whether these systems in turn find relevant sentences that are not found by Ferret. Functionally there are differences across systems. Ferret allows two lists of concepts of unlimited size. Quertle allows large lists (Power Terms™) that cannot be constrained further, Chillibot is limited to 50 entries, Textpresso is limited by species, and both Textpresso and MEDIE require searching one pair at a time. More generally, with Ferret the keyword list does not have to be homogenous. We can, for instance, specify a combination of drugs, GO terms, and diseases in a list if this suits the problem at hand. Chillibot appears to be similar in this regard (though they don’t position their system in this way), while Quertle appears to support only homogenous classes of concepts through their Power Terms™. There are other differences. Quertle for example has the advantage of processing full text by default when possible. Ferret defaults to bibliographic information available in MEDLINE.

**Table 1:** Comparison of systems

	Ferret	Quertle	Textpresso	MEDIE
Brief description	Takes two lists of concepts as input. Retrieves sentences for pairs across two lists and pairs within one list.	Supports semantic searching of concept pairs. A concept may be a single entry or a class of objects via their Power Terms™.	Offers full-text, sentence retrieval from the literature of model organisms or diseases. Although categories of terms such as the cellular component hierarchy of GO may be selected, does not handle a specific list of concepts such as a set of genes.	Retrieves biomedical correlations by specifying pairs of concepts in subject – verb – object structure. Does not handle a list of concepts as input.
Results	Retrieved 38 relevant sentences from 25 relevant PubMed documents referencing 26 species.	Found 1 of 25 relevant documents found by Ferret under focused results.	Found all 15 documents that refer to Arabidopsis. The 25 species referred to by 14 relevant sentences from 10 documents are not indexed by Textpresso.	Found 4 of the 25 relevant documents.

### Case 3: Discoveries related to investigating the role of an AP2 transcription factor gene in Arabidopsis

This case results from a collaboration with Professor Chang and her graduate student Roxane Bouten. Their goal is to investigate the role of a plant-specific Arabidopsis gene that belongs to the AP2 family of transcription factors. They hypothesize that this transcription factor controls plants’ responses to abiotic stresses, such as salinity, drought, cold, and/or wounding, given that other AP2 family members have been shown to regulate gene expression in response to abiotic stress.

They began investigating the function of this putative AP2 transcription factor by designing experiments to determine what effect the overexpression or knock-out of the gene has on Arabidopsis plants. Preliminary experiments showed that plants that overproduce the putative transcription factor display a salt-related phenotype. To gain an understanding of what genes could be involved in this salt response, Bouten conducted a microarray experiment designed to compare gene expression between wild-type plants and plants overexpressing the gene, using mRNA from two-week-old seedlings. Genes that showed increased expression in the microarray are putative downstream targets of the AP2 gene of interest that could be involved in the salt response or other responses.

The results of the microarray experiment returned numerous genes that appear to be differentially regulated in plants overexpressing the gene. Bouten and Chang submitted a list of one hundred genes that exhibited a greater than two-fold (between two- and forty-fold) difference in expression between the overexpressing and wild-type lines. The gene identifiers and a list of 11 keywords (hormone, jasmonate, abscisic acid, cell wall, germination, salt stress, salinity, drought stress, cold stress, wounding, and root) were used by Ferret to mine the literature for evidence of the genes and terms being reported concurrently. The researchers were not interested in gene-gene searches.

The Ferret job began by retrieving 5153 documents for the set of 100 genes. Eleven hundred gene – keyword pair searches were conducted against this set of documents. Twenty-four gene-keyword pair searches returned non-empty results and these found 65 sentences that referenced 48 unique documents. Bouten judged the output from twenty-three searches, finding 15 relevant sentences (from 8 documents) and 41 non-relevant sentences (from 34 documents). One search with 9 sentences was not judged. Precision values are 0.23 for sentences and 0.18 for documents. Although these scores appear to be low, the key point is that the system was able to lead the users to important sentences/documents (describing next) from a starting set of 100 genes, their 5153 documents and 11 keywords.

Bouten and Chang found retrieved sentences that led to research questions they had not been previously considered. For instance, the researchers were not aware of the connection between the gene of interest and salicylic acid. Salicylic acid is a plant hormone that plays a role in defense against pathogens by inducing the production of pathogenesis-related proteins. This could indicate that the putative transcription factor acts in response to biotic as well as abiotic stresses. From other sentences, they learned that the gene could have an effect on downstream targets that inhibit or enhance salt stress responses. This finding supports their hypothesis, since salt stress is a major abiotic stress. Also enumerated in the results was an upregulation of some genes involved in wounding responses, further supporting a possible role in biotic stress response, since pathogens cause wounding of plant tissues. The literature scan performed by Ferret was more research than would have been possible for the investigators to conduct in the absence of Ferret. In addition, because of how the Ferret results are displayed, the researchers could easily view and compare the experimental methods contained within the retrieved literature; this was an unexpected benefit. Bouten and Chang expect that the findings of this research will eventually lead to the ability to manage environmental stresses in crop plants, as climate change and advancing human population put increasing pressures on crop production.

## Conclusions

We presented Ferret a prototype retrieval system designed to find sentences and documents about gene-centric relationships of interest. It is designed to support scientists at different points in their research, from early stage idea exploration to the analysis of large sets of genes related to their lab experiments. Ferret performs cross-species searches and handles gene name ambiguity. We demonstrate its value in three cases. The first case relating to the phenotype of Open Immature Flower is illustrative of early stage research. The second case on ethylene hormone signaling represents more advanced research where the emphasis is on interpreting results from a proteomic study. The third case on potential gene targets involved in plant stress responses represents research at an intermediate level. While success was expressed by the end user bio scientists in all cases comparative performance analysis was done only with case 2. For this case Ferret mined PubMed data to retrieve 38 excellent sentences that pointed to 25 documents from research related to 26 species. Quertle and MEDIE retrieved 1 and 4 of the 25 documents respectively. Textpresso retrieved all 15 of the documents referring to Arabidopsis but not the 10 documents referencing the remaining 25 species. There are design differences in these systems, especially in terms of type of user inputs supported. It is possible though that some of these systems retrieve additional relevant sentences for case 2. This aspect was not assessed due to practical limitations. Ferret does not pay particular attention to negation in a sentence or to the level of confidence (speculation) associated with propositions. These aspects are at the moment left to the user to assess. One option for future research is to explore the merit of automatically identifying and visually highlighting these aspects for the user. Another aspect left to future research relates to query expansion for keywords. Ontologies might be useful in this process; both standardized ones such as Gene Ontology and user-specific ones. Within the constraints of the three cases, this research underlines the importance and contributions of flexible, sentence-based retrieval systems for literature scanning in gene-centric research. We will continue our sentence-based retrieval research by exploring a broader range of case studies.

### Availability of system

Ferret is available to interested bio-scientists. Please contact the first author in order for details. A demo is available at https://mullai.cs.uiowa.edu/ferret3/genesearch/demo/.

## Footnotes

1 http://www.nlm.nih.gov/bsd/index_stats_comp.html#footnote1

2 http://www.nlm.nih.gov/bsd/index_stats_comp.html#footnote1

3 It was confirmed by email communications with Textpresso developers that it is not possible to search multiple species simultaneously.

4 http://www.ncbi.nlm.nih.gov/homologene

5 http://www.nactem.ac.uk/tsujii/GENIA/tagger/

6 http://banner.sourceforge.net/

7 The rating system has recently been extended to allow ratings of (1) unjudged (2) difficult to tell (3) irrelevant (4) good but known (5) interesting (6) excellent.
